# Correction to “Acquired childhood aphasia as a consequence of COVID‐19 and its differential diagnosis from speech–language pathologist perspective: A case study”

**DOI:** 10.1002/ccr3.8256

**Published:** 2023-11-30

Fameen, R, K., RP, S., P, V., R, Bhattarai, B, B. P., A. Acquired childhood aphasia as a consequence of COVID‐19 and its differential diagnosis from speech–language pathologist perspective: A case study. Clin Case Rep. 2022; 10:e06587. doi:10.1002/ccr3.6587

In the article entitled “Acquired childhood aphasia as a consequence of COVID‐19 and its differential diagnosis from speech–language pathologist perspective: A case study” that was previously published in Volume 10 Issue 11 *of Clinical Case Reports*, an author—Mansi Karnad—is added.

The authors list will be:

Ridha Fameen, Rinsha Pravin K., Pooja S., Rashmi V., Biraj Bhattarai, Abhishek B. P., and Mansi Karnad.

We apologize for this error.

